# The Perfluoro Cage Effect: A Search for Electron-Encapsulating Molecules

**DOI:** 10.1021/acsomega.2c07374

**Published:** 2023-01-25

**Authors:** Abhik Ghosh, Jeanet Conradie

**Affiliations:** † Department of Chemistry, UiT − The Arctic University of Norway, Tromsø N-9037, Norway; ‡ Department of Chemistry, University of the Free State, P.O. Box 339, Bloemfontein 9300, South Africa

## Abstract

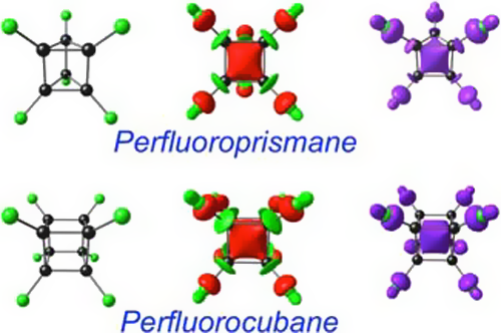

Quantum chemical calculations have for some time predicted that perfluorinated polyhedral organic molecules should exhibit a low-energy LUMO consisting of the overlapping inward-pointing lobes of the C–F σ* orbitals. Accordingly, these molecules should be able to encapsulate an electron within the interior of their cavities. Inspired by the recent confirmation of this prediction for perfluorocubane, we have sought to identify additional perfluorinated cage molecules capable of this remarkable behavior, which we refer to as the perfluoro cage effect (PCE). Using DFT calculations with multiple well-tested exchange-correlation functionals and large STO-QZ4P basis sets, we have identified several systems including [n]prismanes (*n* = 3–6), [n]asteranes (*n* = 3–5), twistane, and two norbornadiene dimer cages that clearly exhibit the PCE. In other words, they exhibit a low-energy LUMO belonging to the total symmetric irreducible representation of the point group in question and adiabatic electron affinities ranging from somewhat under 1 eV to over 2 eV. A pronounced size effect appears to hold, with larger cages exhibiting higher electron affinities (EAs). The largest adiabatic EAs, well over 3 eV, are predicted for perfluorinated dodecahedrane and C_60_. In contrast, the PCE is barely discernible for perfluorinated tetrahedrane and bicyclo[1.1.1]pentane.

## Introduction

Polyfluorination and perfluorination typically affect organic molecules in a profound manner.^1−3^ One such influence is the so-called perfluoro effect, observed for planar conjugated molecules, in which the fluorines exert a much stronger stabilizing influence on the σ molecular orbitals than on the π molecular orbitals.^4−7^ Perfluorinated polyhedral organic molecules have been theoretically examined and a key prediction is a low-energy, totally symmetric LUMO derived from the overlapping inward-pointing lobes of the C–F σ* orbitals.^8,9^ The molecules thus exhibit a significant electron affinity (EA), accommodating the electron largely within central cavity of the polyhedra. This prediction has now been experimentally realized in the form of the perfluorocubane anion radical with *O*_h_ symmetry.^10^ Herein, we have used density functional theory (DFT) to explore both the generality and limitations of the electron-encapsulating effect across a wide range of organofluorine cages. The effect, hereafter referred to as the perfluoro cage effect (PCE), indeed appears to be general, with only a handful of exceptions. Several new examples of the PCE are predicted.

## Results and Discussion

**Figure 1:**
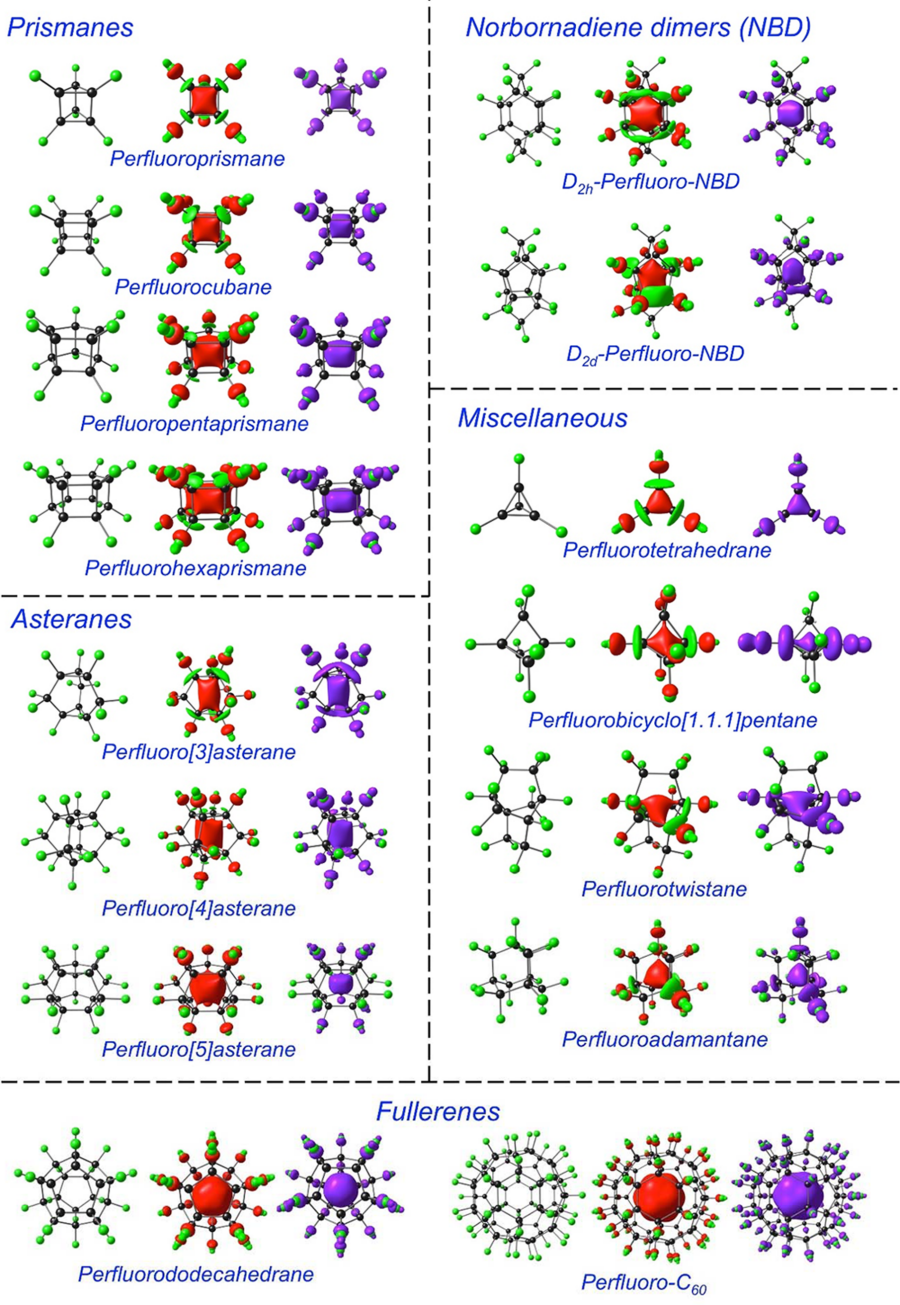
Ball-and-stick structures, neutral LUMOs, anion spin densities of the perfluorinated cage molecules studied.

Sixteen perfluorinated polyhedral and/or cage molecules were examined with three well-tested^11−15^ exchange-correlation functionals, OLYP,^16,17^ B3LYP,^18,19^ and B3LYP*,^20,21^ augmented with D3^22^ dispersion corrections, and large STO-QZ4P basis sets. Table 1 presents their calculated adiabatic EAs, while Figure 1 presents graphical representations of their LUMOs and the spin densities of their anion-radicals. In pretty much every case examined, the LUMO belongs to the totally symmetric irreducible representation of the point group in question. The vast majority of the molecules also exhibit a significant electron-encapsulating ability, as measured by the adiabatic EAs, which, by and large, are mutually consistent across the three functionals.

**Table 1:** Adiabatic Electron Affinities (eV) of the Compounds Studied

			adiabatic EA			
compound	point group	LUMO irrep	OLYP	OLYP (ZPE)	B3LYP*	B3LYP
*Prismanes*						
Perfluoroprismane	*D*_3h_	*a*′_1_	0.97	1.04	0.87	0.80
Octafluorocubane	*O*_h_	*a*_1g_	1.55	1.67	1.43	1.35
Perfluoropentaprismane	*D*_5h_	*a*′_1_	1.98	2.12	1.85	1.77
Perfluorohexaprismane	*D*_6h_	*a*_1g_	2.15	2.28	2.04	1.96
*Asteranes*						
Perfluoro[3]asterane	*C*_3h_	*a*′	1.36	1.54	1.18	1.08
Perfluoro[4]asterane	*D*_4h_	*a*_1g_	2.03	2.19	1.91	1.82
Perfluoro[5]asterane	*D*_5h_	*a*′_1_	2.43	2.59	2.39	2.33
*Perfluoronorbornadiene dimers*						
*D*_2d_-Perfluoro-NBD	*D*_2d_	*a*_1_	2.02	2.18	1.90	1.81
*D*_2h_-Perfluoro-NBD	*D*_2h_	*a*_g_	2.05	2.26	1.96	1.87
*Miscellaneous*						
Perfluorotetrahedrane	*T*_d_	*a*_1_	0.02	0.10	0.07	0.07
Perfluorobicyclo[1.1.1]pentane	*D*_3h_	*a*	–0.03		–0.05	–0.13
Perfluorotwistane	*D*_2_	*a*	0.73	0.99	0.59	0.48
Perfluoroadamantane	*T*_d_	*a*_1_	1.09	1.29	0.93	0.81
*Fullerenes*						
Perfluorododecahedrane	*I*_h_	*a*_1g_	3.44	3.54	3.52	3.46
Perfluoro-C_60_	*I*_h_	*a*_1g_	3.87		4.53	4.50

All the perfluorinated prismanes exhibit sizable electron affinities, with the following order across all three functionals (the values in eV shown within parentheses are for OLYP): hexaprismane^23,24^ (2.15) > pentaprismane^25,26^ (1.98) > cubane^27−29^ (1.55) > prismane^30,31^ (0.97). The ordering suggests a pronounced size effect on the PCE: larger fluorinated cages result in greater stabilization of the encapsulated electron.

The three asteranes^32−36^ examined also exhibit relatively large electron affinities, with that of perfluoro[5]asterane (OLYP: 2.43 eV) and perfluoro[4]asterane (2.03 eV) greatly exceeding that of perfluoro[3]asterane (1.36 eV). Again, there appears to be a pronounced size effect.

The two *T*_d_ systems examined, tetrafluorotetrahedrane^37,38^ and perfluoroadamantane,^39−43^ exhibit dramatically different electron affinities. For the former, the value is near zero, suggesting that the relatively tiny tetrahedral cage cannot effectively encapsulate an electron. The same also holds for perfluorinated bicyclo[1.1.1]pentane.^44,45^ In contrast, perfluorinated twistane^46^ (OLYP: 0.73) and adamantane (1.09 eV) exhibit moderate, positive EAs.

Among the molecules examined here, the largest adiabatic EAs have been calculated for perfluorinated dodecahedrane, C_20_F_20_,^47−49^ and buckministerfullerene, C_60_F_60_,^50−52^ the OLYP values being 3.44 and 3.87 eV, respectively. In each case, the spin density of the anion radical has the shape of a spheroidal shell within the interior of the polyhedral skeletons.

## Concluding Remarks

Given the large number of polyhedral or cage-shaped molecules that have been synthesized and the vastly greater number that are theoretically possible, a clear conclusion from the present study is that electron encapsulation by their perfluorinated counterparts should be widely prevalent – indeed more the rule than the exception – and limited only by the accessibility of the compounds in question. The only exceptions appear to be the smallest cages such as tetrahedrane and bicyclo[1.1.1]pentane.

## Supporting Information Available

Optimized Cartesian coordinates (10 pages) (PDF)

## Notes

The data underlying this study are available in the published article and its online Supplementary Material.

## References

[ref1] Biffinger J. C.; Kim H. W.; DiMagno S. G. The polar hydrophobicity of fluorinated compounds. ChemBioChem 2004, 5, 622–627. 10.1002/cbic.200300910. 15122633

[ref2] Johnson B. M.; Shu Y. Z.; Zhuo X.; Meanwell N. A. Metabolic and pharmaceutical aspects of fluorinated compounds. J. Med. Chem. 2020, 63, 6315–6386. 10.1021/acs.jmedchem.9b01877. 32182061

[ref3] Berger R.; Resnati G.; Metrangolo P.; Weber E.; Hulliger J. Organic fluorine compounds: a great opportunity for enhanced materials properties. Chem. Soc. Rev. 2011, 40, 3496–3508. 10.1039/c0cs00221f. 21448484

[ref4] Brundle C. R.; Robin M. B.; Kuebler N. A.; Basch H. Perfluoro effect in photoelectron spectroscopy. I. Nonaromatic molecules. J. Am. Chem. Soc. 1972, 94, 1451–1465. 10.1021/ja00760a007.

[ref5] Brundle C. R.; Robin M. B.; Kuebler N. A. Perfluoro effect in photoelectron spectroscopy. II. Aromatic molecules. J. Am. Chem. Soc. 1972, 94, 1466–1475. 10.1021/ja00760a008.

[ref6] Van den Ham D. M. W.; Van der Meer D. Perfluoro effect in the photoelectron spectra of quinoline and isoquinoline. Chem. Phys. Lett. 1972, 15, 549–552. 10.1016/0009-2614(72)80368-3.

[ref7] Decleva P.; Stener M.; Holland D. M. P.; Potts A. W.; Karlsson L. Perfluoro effects in the occupied and virtual valence orbitals of hexafluorobenzene. J. Phys. B: At., Mol. Opt. Phys. 2007, 40, 2939 10.1088/0953-4075/40/14/012.

[ref8] Irikura K. K. Sigma stellation: A design strategy for electron boxes. J. Phys. Chem. A 2008, 112, 983–988. 10.1021/jp710372p. 18183966

[ref9] Wang Y. F.; Li Y.; Li Z. R.; Ma F.; Wu D.; Sun C. C. Perfluorinated exohedral potassium-metallofullerene K···C_n_F_n_ (n = 20 or 60): partial interior and surface excess electron state. Theor. Chem. Acc. 2010, 127, 641–650. 10.1007/s00214-010-0763-1.

[ref10] Sugiyama M.; Akiyama M.; Yonezawa Y.; Komaguchi K.; Higashi M.; Nozaki K.; Okazoe T. Electron in a cube: Synthesis and characterization of perfluorocubane as an electron acceptor. Science 2022, 377, 756–759. 10.1126/science.abq0516. 35951682

[ref11] Jacobsen H.; Cavallo L. Re-evaluation of the Mn(salen) mediated epoxidation of alkenes by means of the B3LYP* density functional. Phys. Chem. Chem. Phys. 2004, 6, 3747–3753. 10.1039/b402188f.

[ref12] Conradie M. M.; Conradie J.; Ghosh A. Capturing the spin state diversity of iron(III)-aryl porphyrins OLYP is better than TPSSh. J. Inorg. Biochem. 2011, 105, 84–91. 10.1016/j.jinorgbio.2010.09.010. 21134606

[ref13] Hirao H. Which DFT Functional Performs Well in the Calculation of Methylcobalamin? Comparison of the B3LYP and BP86 Functionals and Evaluation of the Impact of Empirical Dispersion Correction. J. Phys. Chem. A 2011, 115, 9308–9313. 10.1021/jp2052807. 21806069

[ref14] Conradie J.; Ghosh A. DFT Calculations on the Spin-Crossover Complex Fe(salen)(NO): A Quest for the Best Functional. J. Phys. Chem. B 2007, 111, 12621–12624. 10.1021/jp074480t. 17935317

[ref15] Siegbahn P. E. M.; Blomberg M. R. A. A Systematic DFT Approach for Studying Mechanisms of Redox Active Enzymes. Front. Chem. 2018, 6, 644 10.3389/fchem.2018.00644. 30627530 PMC6309562

[ref16] Handy N. C.; Cohen A. J. Left-right correlation energy. Mol. Phys. 2001, 99, 403–412. 10.1080/00268970010018431.

[ref17] Lee C.; Yang W.; Parr R. G. Development of the Colle-Salvetti correlation-energy formula into a functional of the electron density. Phys. Rev. B 1988, 37, 785–789. 10.1103/PhysRevB.37.785. 9944570

[ref18] Becke A. D. Density-functional exchange-energy approximation with correct asymptotic behaviour. Phys. Rev. A 1988, 38, 3098–3100. 10.1103/PhysRevA.38.3098. 9900728

[ref19] Miehlich B.; Savin A.; Stoll H.; Preuss H. Results Obtained with the Correlation Energy Density Functionals of Becke and Lee, Yang and Parr. Chem. Phys. Lett. 1989, 157, 200–206. 10.1016/0009-2614(89)87234-3.

[ref20] Reiher M.; Salomon O.; Hess B. A. Reparameterization of hybrid functionals based on energy differences of states of different multiplicity. Theor. Chem. Acc. 2001, 107, 48–55. 10.1007/s00214-001-0300-3.

[ref21] Salomon O.; Reiher M.; Hess B. A. Assertion and validation of the performance of the B3LYP* functional for the first transition metal row and the G2 test set. J. Chem. Phys. 2002, 117, 4729–4737. 10.1063/1.1493179.

[ref22] Grimme S.; Anthony J.; Ehrlich S.; Krieg H. A Consistent and Accurate *Ab Initio* Parametrization of Density Functional Dispersion Correction (DFT-D) for the 94 Elements H-Pu. J. Chem. Phys. 2010, 132, 154104 10.1063/1.3382344. 20423165

[ref23] Allinger N. L.; Eaton P. E. The geometries of pentaprismane and hexaprismane insights from molecular mechanics. Tetrahedron Lett. 1983, 24, 3697–3700. 10.1016/S0040-4039(00)94512-X.

[ref24] Chou T. C.; Lin G. H.; Yeh Y. L.; Lin K. J. Synthetic Approach Towards Hexaprismane. A Novel Entry to Homosecohexaprismane Skeleton by Cage Enlargement. J. Chin. Chem. Soc. 1997, 44, 477–493. 10.1002/jccs.199700073.

[ref25] Eaton P. E.; Or Y. S.; Branca S. J. Pentaprismane. J. Am. Chem. Soc. 1981, 103, 2134–2136. 10.1021/ja00398a062.

[ref26] Eaton P. E.; Or Y. S.; Branca S. J.; Shankar B. R. The synthesis of pentaprismane. Tetrahedron 1986, 42, 1621–1631. 10.1016/S0040-4020(01)87579-7.

[ref27] Eaton P. E.; Cole T. W. Cubane. J. Am. Chem. Soc. 1964, 86, 3157–3158. 10.1021/ja01069a041.

[ref28] Eaton P. E.; Cole T. W. The cubane system. J. Am. Chem. Soc. 1964, 86, 962–964. 10.1021/ja01059a072.

[ref29] Biegasiewicz K. F.; Griffiths J. R.; Savage G. P.; Tsanaktsidis J.; Priefer R. Cubane: 50 years later. Chem. Rev. 2015, 115, 6719–6745. 10.1021/cr500523x. 26102302

[ref30] Wilzbach K. E.; Kaplan L. Photoisomerization of Tri-*t*-butylbenzenes. Prismane and Benzvalene Isomers. J. Am. Chem. Soc. 1965, 87, 4004–4006. 10.1021/ja01095a055.

[ref31] Katz T. J.; Acton N. Synthesis of prismane. J. Am. Chem. Soc. 1973, 95, 2738–2739. 10.1021/ja00789a084.

[ref32] Biethan U.; Gizycki U. V.; Musso H. Asterane. Tetrahedron Lett. 1965, 6, 477–1482.

[ref33] Musso H. Asterane. Angew. Chem., Int. Ed. 1965, 80, 290–291.

[ref34] Hoffmann V. T.; Musso H. Nonacyclo[10.8.0.0^2,11^.0^4,9^.0^4,19^.0^6,17^.0^7,16^.0^9,14^.0^14,19^]icosan, ein doppeltes Tetraasteran. Angew. Chem., Int. Ed. 1987, 99, 1036–1037. 10.1002/ange.19870991008.

[ref35] Ebel K.; Krüger H.; Musso H. Asterane, XX. Studien in der Pentaasteranreihe. Chem. Ber. 1988, 121, 323–326. 10.1002/cber.19881210219.

[ref36] Bader A.; Ebel K.; Musso H.; Skuballa N. Asterane, XXI. Weitere Versuche zur Synthese des Pentaasterans. Chem. Ber. 1988, 121, 327–338. 10.1002/cber.19881210220.

[ref37] Maier G. Tetrahedrane and cyclobutadiene. Angew. Chem., Int. Ed. 1988, 27, 309–332. 10.1002/anie.198803093.

[ref38] Maier G.; Neudert J.; Wolf O.; Pappusch D.; Sekiguchi A.; Tanaka M.; Matsuo T. Tetrakis(trimethylsilyl)tetrahedrane. J. Am. Chem. Soc. 2002, 124, 13819–13826. 10.1021/ja020863n. 12431112

[ref39] Schleyer P. v. R. A simple preparation of adamantane. J. Am. Chem. Soc. 1957, 79, 3292–3292. 10.1021/ja01569a086.

[ref40] Fort R. C.; Schleyer P. v. R. Adamantane: consequences of the diamondoid structure. Chem. Rev. 1964, 64, 277–300. 10.1021/cr60229a004.

[ref41] Schwertfeger H.; Fokin A. A.; Schreiner P. R. Diamonds are a chemist’s best friend: diamondoid chemistry beyond adamantane. Angew. Chem., Int. Ed. 2008, 47, 1022–1036. 10.1002/anie.200701684. 18081112

[ref42] Robertson G.; Liu E. K. S.; Lagow R. J. Synthesis of perfluoroadamantane compounds by direct fluorination. J. Org. Chem. 1978, 43, 4981–4983. 10.1021/jo00420a020.

[ref43] Li Q. S.; Feng X. J.; Xie Y.; Schaefer H. F. Perfluoroadamantane and its negative ion. J. Phys. Chem. A. 2005, 109, 1454–1457. 10.1021/jp040538h. 16833464

[ref44] Wiberg K. B.; Williams V. Z. Jr. Bicyclo [1.1.1] pentane derivatives. J. Org. Chem. 1970, 35, 369–373. 10.1021/jo00827a018.

[ref45] Kanazawa J.; Uchiyama M. Recent advances in the synthetic chemistry of bicyclo[1.1.1] pentane. Synlett 2019, 30, 1–11. 10.1055/s-0037-1610314.

[ref46] Gauthier J.; Deslongchamps P. A new synthesis of twistane. Can. J. Chem. 1967, 45, 297–300. 10.1139/v67-052.

[ref47] Ternansky R. J.; Balogh D. W.; Paquette L. A. Dodecahedrane. J. Am. Chem. Soc. 1982, 104, 4503–4504. 10.1021/ja00380a040.

[ref48] Paquette L. A.; Ternansky R. J.; Balogh D. W.; Kentgen G. Total synthesis of dodecahedrane. J. Am. Chem. Soc. 1983, 105, 5446–5450. 10.1021/ja00354a043.

[ref49] Schulman J. M.; Disch R. L. Theoretical studies of dodecahedrane. 2. Dodecahedrane, inclusion compounds, and fluorine derivatives. J. Am. Chem. Soc. 1978, 100, 5677–5681. 10.1021/ja00486a016.

[ref50] Ol’ga V. B.; Galeva N. A. Direct fluorination of fullerenes. Russ. Chem. Rev. 2000, 69, 609–621. 10.1070/RC2000v069n07ABEH000579.

[ref51] Holloway J. H.; Hope E. G.; Taylor R.; Langley G. J.; Avent A. G.; Dennis T. J.; Hare J. P.; Kroto H. W.; Walton D. R. Fluorination of buckminsterfullerene. J. Chem. Soc. Chem. Commun. 1991, 966–969. 10.1039/c39910000966.

[ref52] Ravaine S.; Agricole B.; Mingotaud C.; Cousseau J.; Delhaes P. Langmuir and Langmuir-Blodgett films of a perfluoro C_60_ derivative. Chem. Phys. Lett. 1995, 242, 478–482. 10.1016/0009-2614(95)00756-T.

