# Supplementary material for: The Perfluoro Cage Effect: A Search for Electron-Encapsulating Molecules
Source: ACS Omega. 2023 Jan 25;8(5):4972–5. doi: 10.1021/acsomega.2c07374 (PMC9910065; doi:10.1021/acsomega.2c07374)
Supplement: Supplementary file 1 — ao2c07374_si_001.pdf [file ao2c07374_si_001.pdf]

Supporting information

The Perfluoro Cage Effect: A Search For Electron-Encapsulating Molecules

Abhik Ghosh[a,*] and Jeanet Conradie[a,b,*]

[a] Department of Chemistry, UiT – The Arctic University of Norway, N-9037 Tromsø, Norway; Email: abhik.ghosh@uit.no

[b] Department of Chemistry, University of the Free State, P.O. Box 339, Bloemfontein, 9300, South Africa; Email: conradj@ufs.ac.za

Optimized all-electron B3LYP*/STO-QZ4P coordinates (Å)

Table of Contents................ Error! Bookmark not defined.

1. Perfluoroprismane, D_{3h} 2
2. Perfluoroprismane anion, D_{3h} 2
3. Octafluorocubane, O_h 2
4. Octafluorocubane anion, O_h 3
5. Perfluoropentaprismane, D_{5h} 3
6. Perfluoropentaprismane anion, D_{5h} 4
7. Perfluorohexaprismane, D_{6h} 4
8. Perfluorohexaprismane anion, D_{6h} 5
9. Perfluoro[3]asterane, C_{3h} 5
10. Perfluoro[3]asterane anion, C_{3h} 6
11. Perfluoro[4]asterane, D_{4h} 6
12. Perfluoro[4]asterane anion, D_{4h} 7
13. Perfluoro[5]asterane, D_{5h} 8
14. Perfluoro[5]asterane anion, D_{5h} 8
15. D_{2d}-Perfluoro-NBD, D_{2d} 9
16. D_{2d}-Perfluoro-NBD anion, D_{2d} 10
17. D_{2h}-Perfluoro-NBD, D_{2h} 10
18. D_{2h}-Perfluoro-NBD anion, D_{2h} 11
19. Perfluorotetrahedrane, T_d 12
20. Perfluorotetrahedrane anion, T_d 12
21. Perfluorobicyclo[1.1.1]pentane, D_{3h} 12
22. Perfluorobicyclo[1.1.1]pentane anion, D_{3h} 13

23. Perfluorotwistane, D_2 13
24. Perfluorotwistane anion, D_2 13
25. Perfluoroadamantane, T_d 14
26. Perfluoroadamantane anion, T_d 15
27. Perfluorododecahedrane, I_h 15
28. Perfluorododecahedrane anion, I_h 16
29. Perfluoro-C60, I_h 17
30. Perfluoro-C60 anion, I_h 19

1. Perfluoroprismane, D_{3h}

C	0.888494000	0.000000000	0.782446000
C	0.888494000	0.000000000	-0.782446000
C	-0.444247000	0.769459000	0.782446000
C	-0.444247000	0.769459000	-0.782446000
C	-0.444247000	-0.769459000	0.782446000
C	-0.444247000	-0.769459000	-0.782446000
F	1.899088000	0.000000000	1.654471000
F	1.899088000	0.000000000	-1.654471000
F	-0.949544000	1.644658000	1.654471000
F	-0.949544000	1.644658000	-1.654471000
F	-0.949544000	-1.644658000	1.654471000
F	-0.949544000	-1.644658000	-1.654471000

2. Perfluoroprismane anion, D_{3h}

C	0.874908000	0.000000000	0.768334000
C	0.874908000	0.000000000	-0.768334000
C	-0.437454000	0.757692000	0.768334000
C	-0.437454000	0.757692000	-0.768334000
C	-0.437454000	-0.757692000	0.768334000
C	-0.437454000	-0.757692000	-0.768334000
F	1.928844000	0.000000000	1.687495000
F	1.928844000	0.000000000	-1.687495000
F	-0.964422000	1.670428000	1.687495000
F	-0.964422000	1.670428000	-1.687495000
F	-0.964422000	-1.670428000	1.687495000
F	-0.964422000	-1.670428000	-1.687495000

3. Octafluorocubane, O_h

C	0.790032000	0.790032000	0.790032000
C	0.790032000	0.790032000	-0.790032000
C	0.790032000	-0.790032000	0.790032000

C	0.790032000	-0.790032000	-0.790032000
C	-0.790032000	0.790032000	0.790032000
C	-0.790032000	0.790032000	-0.790032000
C	-0.790032000	-0.790032000	0.790032000
C	-0.790032000	-0.790032000	-0.790032000
F	1.563172000	1.563172000	1.563172000
F	1.563172000	1.563172000	-1.563172000
F	1.563172000	-1.563172000	1.563172000
F	1.563172000	-1.563172000	-1.563172000
F	-1.563172000	1.563172000	1.563172000
F	-1.563172000	1.563172000	-1.563172000
F	-1.563172000	-1.563172000	1.563172000
F	-1.563172000	-1.563172000	-1.563172000

4. Octafluorocubane anion, O_h

C	0.778933000	0.778933000	0.778933000
C	0.778933000	0.778933000	-0.778933000
C	0.778933000	-0.778933000	0.778933000
C	0.778933000	-0.778933000	-0.778933000
C	-0.778933000	0.778933000	0.778933000
C	-0.778933000	0.778933000	-0.778933000
C	-0.778933000	-0.778933000	0.778933000
C	-0.778933000	-0.778933000	-0.778933000
F	1.581069000	1.581069000	1.581069000
F	1.581069000	1.581069000	-1.581069000
F	1.581069000	-1.581069000	1.581069000
F	1.581069000	-1.581069000	-1.581069000
F	-1.581069000	1.581069000	1.581069000
F	-1.581069000	1.581069000	-1.581069000
F	-1.581069000	-1.581069000	1.581069000
F	-1.581069000	-1.581069000	-1.581069000

5. Perfluoropentaprismane, D_{5h}

C	1.336655000	0.000000000	-0.788977000
C	1.336655000	0.000000000	0.788977000
C	0.413049000	1.271234000	-0.788977000
C	0.413049000	-1.271234000	-0.788977000
C	0.413049000	-1.271234000	0.788977000
C	0.413049000	1.271234000	0.788977000
C	-1.081377000	0.785666000	-0.788977000
C	-1.081377000	-0.785666000	-0.788977000
C	-1.081377000	-0.785666000	0.788977000
C	-1.081377000	0.785666000	0.788977000
F	0.755606000	-2.325515000	1.547133000
F	-1.978201000	-1.437247000	1.547133000
F	-1.978201000	1.437247000	1.547133000
F	2.445191000	0.000000000	1.547133000

F	0.755606000	2.325515000	1.547133000
F	0.755606000	2.325515000	-1.547133000
F	-1.978201000	1.437247000	-1.547133000
F	-1.978201000	-1.437247000	-1.547133000
F	0.755606000	-2.325515000	-1.547133000
F	2.445191000	0.000000000	-1.547133000

6. Perfluoropentaprismane anion, D_{5h}

C	1.318255000	0.000000000	-0.781056000
C	1.318255000	0.000000000	0.781056000
C	0.407363000	1.253735000	-0.781056000
C	0.407363000	-1.253735000	-0.781056000
C	0.407363000	-1.253735000	0.781056000
C	0.407363000	1.253735000	0.781056000
C	-1.066491000	0.774851000	-0.781056000
C	-1.066491000	-0.774851000	-0.781056000
C	-1.066491000	-0.774851000	0.781056000
C	-1.066491000	0.774851000	0.781056000
F	0.762940000	-2.348086000	1.552179000
F	-1.997402000	-1.451197000	1.552179000
F	-1.997402000	1.451197000	1.552179000
F	2.468924000	0.000000000	1.552179000
F	0.762940000	2.348086000	1.552179000
F	0.762940000	2.348086000	-1.552179000
F	-1.997402000	1.451197000	-1.552179000
F	-1.997402000	-1.451197000	-1.552179000
F	0.762940000	-2.348086000	-1.552179000
F	2.468924000	0.000000000	-1.552179000

7. Perfluorohexaprismane, D_{6h}

C	0.786359000	1.362014000	0.785373000
C	0.786359000	1.362014000	-0.785373000
C	0.786359000	-1.362014000	0.785373000
C	0.786359000	-1.362014000	-0.785373000
C	1.572718000	0.000000000	0.785373000
C	1.572718000	0.000000000	-0.785373000
C	-0.786359000	1.362014000	0.785373000
C	-0.786359000	1.362014000	-0.785373000
C	-0.786359000	-1.362014000	0.785373000
C	-0.786359000	-1.362014000	-0.785373000
C	-1.572718000	0.000000000	0.785373000
C	-1.572718000	0.000000000	-0.785373000
F	1.350804000	2.339661000	1.517472000
F	1.350804000	2.339661000	-1.517472000
F	1.350804000	-2.339661000	1.517472000
F	1.350804000	-2.339661000	-1.517472000

F	2.701608000	0.000000000	1.517472000
F	2.701608000	0.000000000	-1.517472000
F	-1.350804000	2.339661000	1.517472000
F	-1.350804000	2.339661000	-1.517472000
F	-1.350804000	-2.339661000	1.517472000
F	-1.350804000	-2.339661000	-1.517472000
F	-2.701608000	0.000000000	1.517472000
F	-2.701608000	0.000000000	-1.517472000

8. Perfluorohexaprismane anion, D_{6h}

C	0.776781000	1.345424000	0.779578000
C	0.776781000	1.345424000	-0.779578000
C	0.776781000	-1.345424000	0.779578000
C	0.776781000	-1.345424000	-0.779578000
C	1.553562000	0.000000000	0.779578000
C	1.553562000	0.000000000	-0.779578000
C	-0.776781000	1.345424000	0.779578000
C	-0.776781000	1.345424000	-0.779578000
C	-0.776781000	-1.345424000	0.779578000
C	-0.776781000	-1.345424000	-0.779578000
C	-1.553562000	0.000000000	0.779578000
C	-1.553562000	0.000000000	-0.779578000
F	1.363125000	2.361002000	1.511215000
F	1.363125000	2.361002000	-1.511215000
F	1.363125000	-2.361002000	1.511215000
F	1.363125000	-2.361002000	-1.511215000
F	2.726250000	0.000000000	1.511215000
F	2.726250000	0.000000000	-1.511215000
F	-1.363125000	2.361002000	1.511215000
F	-1.363125000	2.361002000	-1.511215000
F	-1.363125000	-2.361002000	1.511215000
F	-1.363125000	-2.361002000	-1.511215000
F	-2.726250000	0.000000000	1.511215000
F	-2.726250000	0.000000000	-1.511215000

9. Perfluoro[3]asterane, C_{3h}

C	-1.736740000	0.000000000	0.000000000
C	-0.878654000	0.000000000	1.252266000
C	0.439327000	-0.760937000	1.252266000
C	-0.878654000	0.000000000	-1.252266000
C	0.439327000	0.760937000	-1.252266000
C	0.868370000	1.504061000	0.000000000
C	0.439327000	0.760937000	1.252266000
C	0.868370000	-1.504061000	0.000000000
C	0.439327000	-0.760937000	-1.252266000

F	0.793222000	1.373901000	2.393436000
F	0.793222000	-1.373901000	2.393436000
F	-1.586444000	0.000000000	2.393436000
F	0.793222000	-1.373901000	-2.393436000
F	0.321847000	2.749851000	0.000000000
F	2.220517000	1.653653000	0.000000000
F	-2.542365000	1.096198000	0.000000000
F	-2.542365000	-1.096198000	0.000000000
F	0.321847000	-2.749851000	0.000000000
F	2.220517000	-1.653653000	0.000000000
F	0.793222000	1.373901000	-2.393436000
F	-1.586444000	0.000000000	-2.393436000

10. Perfluoro[3]asterane anion, C_{3h}

C	-1.736515000	0.000000000	0.000000000
C	-0.865016000	0.000000000	1.222683000
C	0.432508000	-0.749126000	1.222683000
C	-0.865016000	0.000000000	-1.222683000
C	0.432508000	0.749126000	-1.222683000
C	0.868258000	1.503866000	0.000000000
C	0.432508000	0.749126000	1.222683000
C	0.868258000	-1.503866000	0.000000000
C	0.432508000	-0.749126000	-1.222683000
F	0.804644000	1.393685000	2.417923000
F	0.804644000	-1.393685000	2.417923000
F	-1.609289000	0.000000000	2.417923000
F	0.804644000	-1.393685000	-2.417923000
F	0.327617000	2.772651000	0.000000000
F	2.237378000	1.670050000	0.000000000
F	-2.564995000	1.102601000	0.000000000
F	-2.564995000	-1.102601000	0.000000000
F	0.327617000	-2.772651000	0.000000000
F	2.237378000	-1.670050000	0.000000000
F	0.804644000	1.393685000	-2.417923000
F	-1.609289000	0.000000000	-2.417923000

11. Perfluoro[4]asterane, D_{4h}

C	0.000000000	2.028445000	0.000000000
C	-2.028445000	0.000000000	0.000000000
C	0.000000000	1.122390000	-1.247737000
C	-1.122390000	0.000000000	-1.247737000
C	0.000000000	1.122390000	1.247737000
C	1.122390000	0.000000000	-1.247737000
C	-1.122390000	0.000000000	1.247737000
C	0.000000000	-1.122390000	1.247737000
C	0.000000000	-2.028445000	0.000000000
C	0.000000000	-1.122390000	-1.247737000

C	2.028445000	0.000000000	0.000000000
C	1.122390000	0.000000000	1.247737000
F	0.000000000	-1.863278000	-2.374350000
F	1.863278000	0.000000000	-2.374350000
F	-1.863278000	0.000000000	-2.374350000
F	0.000000000	1.863278000	-2.374350000
F	0.000000000	1.863278000	2.374350000
F	1.863278000	0.000000000	2.374350000
F	-1.079619000	-2.843140000	0.000000000
F	1.079619000	-2.843140000	0.000000000
F	-2.843140000	-1.079619000	0.000000000
F	-2.843140000	1.079619000	0.000000000
F	1.079619000	2.843140000	0.000000000
F	-1.079619000	2.843140000	0.000000000
F	2.843140000	1.079619000	0.000000000
F	2.843140000	-1.079619000	0.000000000
F	0.000000000	-1.863278000	2.374350000
F	-1.863278000	0.000000000	2.374350000

12. Perfluoro[4]asterane anion, D_{4h}

C	0.000000000	2.021145000	0.000000000
C	-2.021145000	0.000000000	0.000000000
C	0.000000000	1.103216000	-1.228144000
C	-1.103216000	0.000000000	-1.228144000
C	0.000000000	1.103216000	1.228144000
C	1.103216000	0.000000000	-1.228144000
C	-1.103216000	0.000000000	1.228144000
C	0.000000000	-1.103216000	1.228144000
C	0.000000000	-2.021145000	0.000000000
C	0.000000000	-1.103216000	-1.228144000
C	2.021145000	0.000000000	0.000000000
C	1.103216000	0.000000000	1.228144000
F	0.000000000	-1.890379000	-2.384253000
F	1.890379000	0.000000000	-2.384253000
F	-1.890379000	0.000000000	-2.384253000
F	0.000000000	1.890379000	-2.384253000
F	0.000000000	1.890379000	2.384253000
F	1.890379000	0.000000000	2.384253000
F	-1.081415000	-2.853870000	0.000000000
F	1.081415000	-2.853870000	0.000000000
F	-2.853870000	-1.081415000	0.000000000
F	-2.853870000	1.081415000	0.000000000
F	1.081415000	2.853870000	0.000000000
F	-1.081415000	2.853870000	0.000000000
F	2.853870000	1.081415000	0.000000000
F	2.853870000	-1.081415000	0.000000000
F	0.000000000	-1.890379000	2.384253000
F	-1.890379000	0.000000000	2.384253000

13. Perfluoro[5]asterane, D_{5h}

C	1.095014000	0.795574000	1.220508000
C	1.095014000	0.795574000	-1.220508000
C	1.095014000	-0.795574000	1.220508000
C	1.095014000	-0.795574000	-1.220508000
C	1.894338000	1.376317000	0.000000000
C	1.894338000	-1.376317000	0.000000000
C	-0.418258000	1.287266000	1.220508000
C	-0.418258000	1.287266000	-1.220508000
C	-0.418258000	-1.287266000	1.220508000
C	-0.418258000	-1.287266000	-1.220508000
C	-0.723573000	2.226927000	0.000000000
C	-0.723573000	-2.226927000	0.000000000
C	-1.353512000	0.000000000	1.220508000
C	-1.353512000	0.000000000	-1.220508000
C	-2.341530000	0.000000000	0.000000000
F	1.722214000	1.251262000	2.375545000
F	1.722214000	1.251262000	-2.375545000
F	1.722214000	-1.251262000	2.375545000
F	1.722214000	-1.251262000	-2.375545000
F	2.010391000	2.726203000	0.000000000
F	2.010391000	-2.726203000	0.000000000
F	3.214018000	1.069553000	0.000000000
F	3.214018000	-1.069553000	0.000000000
F	-0.024019000	3.387223000	0.000000000
F	-0.024019000	-3.387223000	0.000000000
F	-0.657827000	2.024584000	2.375545000
F	-0.657827000	2.024584000	-2.375545000
F	-0.657827000	-2.024584000	2.375545000
F	-0.657827000	-2.024584000	-2.375545000
F	-1.971528000	2.754439000	0.000000000
F	-1.971528000	-2.754439000	0.000000000
F	-2.128774000	0.000000000	2.375545000
F	-2.128774000	0.000000000	-2.375545000
F	-3.228862000	1.023866000	0.000000000
F	-3.228862000	-1.023866000	0.000000000

14. Perfluoro[5]asterane anion, D_{5h}

C	1.118266000	0.812468000	1.229867000
C	1.118266000	0.812468000	-1.229867000
C	1.118266000	-0.812468000	1.229867000
C	1.118266000	-0.812468000	-1.229867000
C	1.911209000	1.388574000	0.000000000
C	1.911209000	-1.388574000	0.000000000
C	-0.427140000	1.314601000	1.229867000
C	-0.427140000	1.314601000	-1.229867000
C	-0.427140000	-1.314601000	1.229867000
C	-0.427140000	-1.314601000	-1.229867000

C	-0.730017000	2.246761000	0.000000000
C	-0.730017000	-2.246761000	0.000000000
C	-1.382253000	0.000000000	1.229867000
C	-1.382253000	0.000000000	-1.229867000
C	-2.362384000	0.000000000	0.000000000
F	1.700770000	1.235682000	2.372422000
F	1.700770000	1.235682000	-2.372422000
F	1.700770000	-1.235682000	2.372422000
F	1.700770000	-1.235682000	-2.372422000
F	2.012469000	2.729276000	0.000000000
F	2.012469000	-2.729276000	0.000000000
F	3.217583000	1.070579000	0.000000000
F	3.217583000	-1.070579000	0.000000000
F	-0.023893000	3.390930000	0.000000000
F	-0.023893000	-3.390930000	0.000000000
F	-0.649636000	1.999375000	2.372422000
F	-0.649636000	1.999375000	-2.372422000
F	-0.649636000	-1.999375000	2.372422000
F	-0.649636000	-1.999375000	-2.372422000
F	-1.973809000	2.757364000	0.000000000
F	-1.973809000	-2.757364000	0.000000000
F	-2.102267000	0.000000000	2.372422000
F	-2.102267000	0.000000000	-2.372422000
F	-3.232350000	1.025132000	0.000000000
F	-3.232350000	-1.025132000	0.000000000

15. D_{2d}-Perfluoro-NBD, D_{2d}

C	0.276798000	-1.401836000	-0.723274000
C	-0.806966000	-0.806966000	-1.659593000
C	-1.401836000	0.276798000	-0.723274000
C	-1.401836000	-0.276798000	0.723274000
C	-0.276798000	-1.401836000	0.723274000
C	0.806966000	-0.806966000	1.659593000
C	0.000000000	0.000000000	2.698833000
C	-0.806966000	0.806966000	1.659593000
C	0.276798000	1.401836000	0.723274000
C	1.401836000	0.276798000	0.723274000
C	-0.276798000	1.401836000	-0.723274000
C	0.806966000	0.806966000	-1.659593000
C	1.401836000	-0.276798000	-0.723274000
C	0.000000000	0.000000000	-2.698833000
F	-2.600435000	0.754143000	-1.119085000
F	0.754143000	-2.600435000	-1.119085000
F	-0.754143000	2.600435000	-1.119085000
F	2.600435000	-0.754143000	-1.119085000
F	-1.700092000	-1.700092000	-2.138533000
F	1.700092000	1.700092000	-2.138533000
F	1.700092000	-1.700092000	2.138533000
F	-1.700092000	1.700092000	2.138533000

F	-2.600435000	-0.754143000	1.119085000
F	-0.754143000	-2.600435000	1.119085000
F	0.754143000	2.600435000	1.119085000
F	2.600435000	0.754143000	1.119085000
F	-0.773345000	0.773345000	-3.489000000
F	0.773345000	-0.773345000	-3.489000000
F	0.773345000	0.773345000	3.489000000
F	-0.773345000	-0.773345000	3.489000000

16. D_{2d}-Perfluoro-NBD anion, D_{2d}

C	0.283830000	-1.387784000	-0.714893000
C	-0.798642000	-0.798642000	-1.645224000
C	-1.387784000	0.283830000	-0.714893000
C	-1.387784000	-0.283830000	0.714893000
C	-0.283830000	-1.387784000	0.714893000
C	0.798642000	-0.798642000	1.645224000
C	0.000000000	0.000000000	2.680113000
C	-0.798642000	0.798642000	1.645224000
C	0.283830000	1.387784000	0.714893000
C	1.387784000	0.283830000	0.714893000
C	-0.283830000	1.387784000	-0.714893000
C	0.798642000	0.798642000	-1.645224000
C	1.387784000	-0.283830000	-0.714893000
C	0.000000000	0.000000000	-2.680113000
F	-2.644710000	0.733431000	-1.122444000
F	0.733431000	-2.644710000	-1.122444000
F	-0.733431000	2.644710000	-1.122444000
F	2.644710000	-0.733431000	-1.122444000
F	-1.697466000	-1.697466000	-2.158164000
F	1.697466000	1.697466000	-2.158164000
F	1.697466000	-1.697466000	2.158164000
F	-1.697466000	1.697466000	2.158164000
F	-2.644710000	-0.733431000	1.122444000
F	-0.733431000	-2.644710000	1.122444000
F	0.733431000	2.644710000	1.122444000
F	2.644710000	0.733431000	1.122444000
F	-0.771853000	0.771853000	-3.489225000
F	0.771853000	-0.771853000	-3.489225000
F	0.771853000	0.771853000	3.489225000
F	-0.771853000	-0.771853000	3.489225000

17. D_{2h}-Perfluoro-NBD, D_{2h}

C	-1.150941000	0.000000000	-1.582692000
C	-1.150941000	0.000000000	1.582692000
C	-0.781303000	-1.252082000	-0.773186000
C	-0.781303000	-1.252082000	0.773186000
C	-0.781303000	1.252082000	-0.773186000

C	0.781303000	-1.252082000	-0.773186000
C	-0.781303000	1.252082000	0.773186000
C	0.781303000	1.252082000	0.773186000
C	1.150941000	0.000000000	1.582692000
C	0.781303000	-1.252082000	0.773186000
C	1.150941000	0.000000000	-1.582692000
C	0.781303000	1.252082000	-0.773186000
F	1.402677000	-2.379084000	1.308430000
F	1.402677000	-2.379084000	-1.308430000
F	-1.402677000	-2.379084000	1.308430000
F	-1.402677000	-2.379084000	-1.308430000
F	-1.402677000	2.379084000	-1.308430000
F	1.402677000	2.379084000	-1.308430000
C	0.000000000	0.000000000	2.633445000
C	0.000000000	0.000000000	-2.633445000
F	1.402677000	2.379084000	1.308430000
F	-1.402677000	2.379084000	1.308430000
F	0.000000000	1.087029000	3.441913000
F	0.000000000	-1.087029000	3.441913000
F	0.000000000	1.087029000	-3.441913000
F	0.000000000	-1.087029000	-3.441913000
F	-2.418623000	0.000000000	2.117215000
F	2.418623000	0.000000000	2.117215000
F	-2.418623000	0.000000000	-2.117215000
F	2.418623000	0.000000000	-2.117215000

18. D_{2h}-Perfluoro-NBD anion, D_{2h}

C	-1.153812000	0.000000000	-1.589133000
C	-1.153812000	0.000000000	1.589133000
C	-0.790633000	-1.267955000	-0.783098000
C	-0.790633000	-1.267955000	0.783098000
C	-0.790633000	1.267955000	-0.783098000
C	0.790633000	-1.267955000	-0.783098000
C	-0.790633000	1.267955000	0.783098000
C	0.790633000	1.267955000	0.783098000
C	1.153812000	0.000000000	1.589133000
C	0.790633000	-1.267955000	0.783098000
C	1.153812000	0.000000000	-1.589133000
C	0.790633000	1.267955000	-0.783098000
F	1.374734000	-2.365398000	1.309002000
F	1.374734000	-2.365398000	-1.309002000
F	-1.374734000	-2.365398000	1.309002000
F	-1.374734000	-2.365398000	-1.309002000
F	-1.374734000	2.365398000	-1.309002000
F	1.374734000	2.365398000	-1.309002000
C	0.000000000	0.000000000	2.652255000
C	0.000000000	0.000000000	-2.652255000
F	1.374734000	2.365398000	1.309002000
F	-1.374734000	2.365398000	1.309002000

F	0.000000000	1.089542000	3.442671000
F	0.000000000	-1.089542000	3.442671000
F	0.000000000	1.089542000	-3.442671000
F	0.000000000	-1.089542000	-3.442671000
F	-2.418314000	0.000000000	2.066329000
F	2.418314000	0.000000000	2.066329000
F	-2.418314000	0.000000000	-2.066329000
F	2.418314000	0.000000000	-2.066329000

19. Perfluorotetrahedrane, T_d

C	0.529330000	0.529330000	-0.529330000
C	0.529330000	-0.529330000	0.529330000
C	-0.529330000	0.529330000	0.529330000
C	-0.529330000	-0.529330000	-0.529330000
F	1.293787000	1.293787000	-1.293787000
F	1.293787000	-1.293787000	1.293787000
F	-1.293787000	1.293787000	1.293787000
F	-1.293787000	-1.293787000	-1.293787000

20. Perfluorotetrahedrane anion, T_d

C	0.521236000	0.521236000	-0.521236000
C	0.521236000	-0.521236000	0.521236000
C	-0.521236000	0.521236000	0.521236000
C	-0.521236000	-0.521236000	-0.521236000
F	1.320668000	1.320668000	-1.320668000
F	1.320668000	-1.320668000	1.320668000
F	-1.320668000	1.320668000	1.320668000
F	-1.320668000	-1.320668000	-1.320668000

21. Perfluorobicyclo[1.1.1]pentane, D_{3h}

C	0.000000000	0.000000000	-0.962922000
C	-0.621628000	-1.076690000	0.000000000
C	0.000000000	0.000000000	0.962922000
C	-0.621628000	1.076690000	0.000000000
C	1.243255000	0.000000000	0.000000000
F	-0.093169000	-2.311063000	0.000000000
F	-1.954854000	-1.236218000	0.000000000
F	-0.093169000	2.311063000	0.000000000
F	-1.954854000	1.236218000	0.000000000
F	0.000000000	0.000000000	-2.292875000
F	0.000000000	0.000000000	2.292875000
F	2.048024000	1.074844000	0.000000000
F	2.048024000	-1.074844000	0.000000000

22. Perfluorobicyclo[1.1.1]pentane anion, D_{3h}

C	0.000000000	0.000000000	-0.949604000
C	-0.609775000	-1.056161000	0.000000000
C	0.000000000	0.000000000	0.949604000
C	-0.609775000	1.056161000	0.000000000
C	1.219549000	0.000000000	0.000000000
F	-0.090203000	-2.310221000	0.000000000
F	-1.955608000	-1.233228000	0.000000000
F	-0.090203000	2.310221000	0.000000000
F	-1.955608000	1.233228000	0.000000000
F	0.000000000	0.000000000	-2.560383000
F	0.000000000	0.000000000	2.560383000
F	2.045811000	1.076992000	0.000000000
F	2.045811000	-1.076992000	0.000000000

23. Perfluorotwistane, D_2

C	0.488138000	-0.645709000	1.126141000
C	-0.488138000	0.645709000	1.126141000
C	-0.488138000	-0.645709000	-1.126141000
C	0.488138000	0.645709000	-1.126141000
C	0.000000000	-1.619272000	0.000000000
C	0.000000000	1.619272000	0.000000000
C	1.964087000	-0.257611000	0.757175000
C	-1.964087000	0.257611000	0.757175000
C	1.964087000	0.257611000	-0.757175000
C	-1.964087000	-0.257611000	-0.757175000
F	0.458976000	-1.230075000	2.346076000
F	-0.458976000	1.230075000	2.346076000
F	-0.458976000	-1.230075000	-2.346076000
F	0.458976000	1.230075000	-2.346076000
F	0.965185000	-2.434388000	-0.466572000
F	-0.965185000	-2.434388000	0.466572000
F	-0.965185000	2.434388000	-0.466572000
F	0.965185000	2.434388000	0.466572000
F	2.418067000	0.694038000	1.594937000
F	2.776128000	-1.320522000	0.896935000
F	-2.418067000	-0.694038000	1.594937000
F	-2.776128000	1.320522000	0.896935000
F	2.418067000	-0.694038000	-1.594937000
F	2.776128000	1.320522000	-0.896935000
F	-2.418067000	0.694038000	-1.594937000
F	-2.776128000	-1.320522000	-0.896935000

24. Perfluorotwistane anion, D_2

C	0.471356000	-0.624584000	1.105514000

C	-0.471356000	0.624584000	1.105514000
C	-0.471356000	-0.624584000	-1.105514000
C	0.471356000	0.624584000	-1.105514000
C	0.000000000	-1.609730000	0.000000000
C	0.000000000	1.609730000	0.000000000
C	1.937463000	-0.251128000	0.749492000
C	-1.937463000	0.251128000	0.749492000
C	1.937463000	0.251128000	-0.749492000
C	-1.937463000	-0.251128000	-0.749492000
F	0.494684000	-1.270235000	2.405047000
F	-0.494684000	1.270235000	2.405047000
F	-0.494684000	-1.270235000	-2.405047000
F	0.494684000	1.270235000	-2.405047000
F	0.978636000	-2.444832000	-0.449598000
F	-0.978636000	-2.444832000	0.449598000
F	-0.978636000	2.444832000	-0.449598000
F	0.978636000	2.444832000	0.449598000
F	2.405645000	0.704085000	1.587695000
F	2.765139000	-1.318408000	0.901856000
F	-2.405645000	-0.704085000	1.587695000
F	-2.765139000	1.318408000	0.901856000
F	2.405645000	-0.704085000	-1.587695000
F	2.765139000	1.318408000	-0.901856000
F	-2.405645000	0.704085000	-1.587695000
F	-2.765139000	-1.318408000	-0.901856000

25. Perfluoroadamantane, T_d

C	0.000000000	0.000000000	1.806540000
C	0.904072000	-0.904072000	0.904072000
C	0.000000000	-1.806540000	0.000000000
C	-0.904072000	-0.904072000	-0.904072000
C	0.000000000	0.000000000	-1.806540000
C	0.904072000	0.904072000	-0.904072000
C	0.000000000	1.806540000	0.000000000
C	-0.904072000	0.904072000	0.904072000
C	1.806540000	0.000000000	0.000000000
C	-1.806540000	0.000000000	0.000000000
F	-0.763109000	-2.613538000	0.763109000
F	0.763109000	-2.613538000	-0.763109000
F	-1.688101000	-1.688101000	-1.688101000
F	-2.613538000	-0.763109000	0.763109000
F	-2.613538000	0.763109000	-0.763109000
F	-0.763109000	0.763109000	-2.613538000
F	0.763109000	-0.763109000	-2.613538000
F	1.688101000	1.688101000	-1.688101000
F	0.763109000	2.613538000	0.763109000
F	-0.763109000	2.613538000	-0.763109000
F	-1.688101000	1.688101000	1.688101000
F	0.763109000	0.763109000	2.613538000

F	-0.763109000	-0.763109000	2.613538000
F	1.688101000	-1.688101000	1.688101000
F	2.613538000	0.763109000	0.763109000
F	2.613538000	-0.763109000	-0.763109000

26. Perfluoroadamantane anion, T_d

C	0.000000000	0.000000000	1.789241000
C	0.887923000	-0.887923000	0.887923000
C	0.000000000	-1.789241000	0.000000000
C	-0.887923000	-0.887923000	-0.887923000
C	0.000000000	0.000000000	-1.789241000
C	0.887923000	0.887923000	-0.887923000
C	0.000000000	1.789241000	0.000000000
C	-0.887923000	0.887923000	0.887923000
C	1.789241000	0.000000000	0.000000000
C	-1.789241000	0.000000000	0.000000000
F	-0.767007000	-2.613806000	0.767007000
F	0.767007000	-2.613806000	-0.767007000
F	-1.728300000	-1.728300000	-1.728300000
F	-2.613806000	-0.767007000	0.767007000
F	-2.613806000	0.767007000	-0.767007000
F	-0.767007000	0.767007000	-2.613806000
F	0.767007000	-0.767007000	-2.613806000
F	1.728300000	1.728300000	-1.728300000
F	0.767007000	2.613806000	0.767007000
F	-0.767007000	2.613806000	-0.767007000
F	-1.728300000	1.728300000	1.728300000
F	0.767007000	0.767007000	2.613806000
F	-0.767007000	-0.767007000	2.613806000
F	1.728300000	-1.728300000	1.728300000
F	2.613806000	0.767007000	0.767007000
F	2.613806000	-0.767007000	-0.767007000

27. Perfluorododecahedrane, I_h

C	2.047450000	0.665257000	0.411123000
C	1.265394000	1.741665000	-0.411123000
C	2.047450000	-0.665257000	-0.411123000
C	1.265497000	0.411185000	1.741817000
C	1.265497000	-0.411185000	-1.741817000
C	0.782120000	1.076496000	-1.741817000
C	0.000000000	-1.330622000	-1.741817000
C	1.265394000	-1.741665000	0.411123000
C	-1.265497000	-0.411185000	-1.741817000
C	-0.782120000	1.076496000	-1.741817000

C	-2.047450000	-0.665257000	-0.411123000
C	0.000000000	-2.152816000	-0.411123000
C	-2.047450000	0.665257000	0.411123000
C	-1.265394000	1.741665000	-0.411123000
C	-1.265497000	0.411185000	1.741817000
C	-1.265394000	-1.741665000	0.411123000
C	0.000000000	2.152816000	0.411123000
C	0.000000000	1.330622000	1.741817000
C	0.782120000	-1.076496000	1.741817000
C	-0.782120000	-1.076496000	1.741817000
F	0.000000000	-2.153133000	-2.817004000
F	-2.047752000	-0.665355000	-2.817004000
F	-1.265580000	1.741921000	-2.817004000
F	-2.046801000	2.817180000	-0.665780000
F	0.000000000	3.482226000	0.665780000
F	0.000000000	2.153133000	2.817004000
F	2.047752000	0.665355000	2.817004000
F	1.265580000	-1.741921000	2.817004000
F	2.046801000	-2.817180000	0.665780000
F	0.000000000	-3.482226000	-0.665780000
F	-2.046801000	-2.817180000	0.665780000
F	-3.311794000	-1.076067000	-0.665780000
F	-3.311794000	1.076067000	0.665780000
F	-2.047752000	0.665355000	2.817004000
F	-1.265580000	-1.741921000	2.817004000
F	2.047752000	-0.665355000	-2.817004000
F	3.311794000	-1.076067000	-0.665780000
F	3.311794000	1.076067000	0.665780000
F	2.046801000	2.817180000	-0.665780000
F	1.265580000	1.741921000	-2.817004000

28. Perfluorododecahedrane anion, I_h

C	2.036042000	0.661550000	0.408763000
C	1.258343000	1.731961000	-0.408763000
C	2.036042000	-0.661550000	-0.408763000
C	1.258380000	0.408872000	1.731545000
C	1.258380000	-0.408872000	-1.731545000
C	0.777721000	1.070442000	-1.731545000
C	0.000000000	-1.323139000	-1.731545000
C	1.258343000	-1.731961000	0.408763000
C	-1.258380000	-0.408872000	-1.731545000
C	-0.777721000	1.070442000	-1.731545000
C	-2.036042000	-0.661550000	-0.408763000
C	0.000000000	-2.140821000	-0.408763000
C	-2.036042000	0.661550000	0.408763000
C	-1.258343000	1.731961000	-0.408763000
C	-1.258380000	0.408872000	1.731545000
C	-1.258343000	-1.731961000	0.408763000
C	0.000000000	2.140821000	0.408763000

C	0.000000000	1.323139000	1.731545000
C	0.777721000	-1.070442000	1.731545000
C	-0.777721000	-1.070442000	1.731545000
F	0.000000000	-2.158247000	-2.825113000
F	-2.052615000	-0.666935000	-2.825113000
F	-1.268586000	1.746058000	-2.825113000
F	-2.052508000	2.825035000	-0.669083000
F	0.000000000	3.491935000	0.669083000
F	0.000000000	2.158247000	2.825113000
F	2.052615000	0.666935000	2.825113000
F	1.268586000	-1.746058000	2.825113000
F	2.052508000	-2.825035000	0.669083000
F	0.000000000	-3.491935000	-0.669083000
F	-2.052508000	-2.825035000	0.669083000
F	-3.321028000	-1.079067000	-0.669083000
F	-3.321028000	1.079067000	0.669083000
F	-2.052615000	0.666935000	2.825113000
F	-1.268586000	-1.746058000	2.825113000
F	2.052615000	-0.666935000	-2.825113000
F	3.321028000	-1.079067000	-0.669083000
F	3.321028000	1.079067000	0.669083000
F	2.052508000	2.825035000	-0.669083000
F	1.268586000	1.746058000	-2.825113000

29. Perfluoro-C60, I_h

C	0.000000000	1.382079000	-3.777998000
C	0.000000000	2.761439000	-2.925573000
C	0.000000000	-1.382079000	3.777998000
C	0.000000000	-2.761439000	2.925573000
C	0.810818000	3.352367000	2.071714000
C	0.810818000	-3.352367000	-2.071714000
C	0.812366000	1.118125000	3.777998000
C	0.812366000	-1.118125000	-3.777998000
C	0.812495000	3.879781000	-0.689623000
C	0.812495000	-3.879781000	0.689623000
C	1.314435000	0.427086000	-3.777998000
C	1.314435000	-0.427086000	3.777998000
C	1.314506000	3.188709000	-2.071714000
C	1.314506000	-3.188709000	2.071714000
C	1.623133000	2.234051000	2.925573000
C	1.623133000	-2.234051000	-2.925573000
C	1.623156000	3.616381000	0.689623000
C	1.623156000	-3.616381000	-0.689623000
C	2.626284000	0.853332000	-2.925573000
C	2.626284000	-0.853332000	2.925573000
C	2.626438000	2.235535000	-2.071714000
C	2.626438000	-2.235535000	2.071714000
C	2.937734000	1.807072000	2.071714000
C	2.937734000	-1.807072000	-2.071714000

C	2.937800000	2.661236000	0.689623000
C	2.937800000	-2.661236000	-0.689623000
C	3.438816000	1.971647000	-0.689623000
C	3.438816000	-1.971647000	0.689623000
C	3.438848000	0.264805000	2.071714000
C	3.438848000	-0.264805000	-2.071714000
C	3.940966000	0.426190000	-0.689623000
C	3.940966000	-0.426190000	0.689623000
C	-0.810818000	3.352367000	2.071714000
C	-0.810818000	-3.352367000	-2.071714000
C	-0.812366000	1.118125000	3.777998000
C	-0.812366000	-1.118125000	-3.777998000
C	-0.812495000	3.879781000	-0.689623000
C	-0.812495000	-3.879781000	0.689623000
C	-1.314435000	0.427086000	-3.777998000
C	-1.314435000	-0.427086000	3.777998000
C	-1.314506000	3.188709000	-2.071714000
C	-1.314506000	-3.188709000	2.071714000
C	-1.623133000	2.234051000	2.925573000
C	-1.623133000	-2.234051000	-2.925573000
C	-1.623156000	3.616381000	0.689623000
C	-1.623156000	-3.616381000	-0.689623000
C	-2.626284000	0.853332000	-2.925573000
C	-2.626284000	-0.853332000	2.925573000
C	-2.626438000	2.235535000	-2.071714000
C	-2.626438000	-2.235535000	2.071714000
C	-2.937734000	1.807072000	2.071714000
C	-2.937734000	-1.807072000	-2.071714000
C	-2.937800000	2.661236000	0.689623000
C	-2.937800000	-2.661236000	-0.689623000
C	-3.438816000	1.971647000	-0.689623000
C	-3.438816000	-1.971647000	0.689623000
C	-3.438848000	0.264805000	2.071714000
C	-3.438848000	-0.264805000	-2.071714000
C	-3.940966000	0.426190000	-0.689623000
C	-3.940966000	-0.426190000	0.689623000
F	0.000000000	1.857631000	-5.050848000
F	0.000000000	3.687093000	-3.920073000
F	0.000000000	-1.857631000	5.050848000
F	0.000000000	-3.687093000	3.920073000
F	1.075506000	4.485854000	2.772510000
F	1.075506000	-4.485854000	-2.772510000
F	1.091888000	1.502855000	5.050848000
F	1.091888000	-1.502855000	-5.050848000
F	1.092029000	5.190111000	-0.915060000
F	1.092029000	-5.190111000	0.915060000
F	1.766616000	4.261299000	-2.772510000
F	1.766616000	-4.261299000	2.772510000
F	1.766712000	0.574039000	-5.050848000
F	1.766712000	-0.574039000	5.050848000
F	2.167201000	4.840767000	0.915060000

F	2.167201000	-4.840767000	-0.915060000
F	2.167219000	2.982921000	3.920073000
F	2.167219000	-2.982921000	-3.920073000
F	3.506634000	1.139374000	-3.920073000
F	3.506634000	-1.139374000	3.920073000
F	3.506822000	2.996965000	-2.772510000
F	3.506822000	-2.996965000	2.772510000
F	3.933951000	2.409072000	2.772510000
F	3.933951000	-2.409072000	-2.772510000
F	3.934141000	3.557010000	0.915060000
F	3.934141000	-3.557010000	-0.915060000
F	4.598634000	2.642414000	-0.915060000
F	4.598634000	-2.642414000	0.915060000
F	4.598650000	0.363338000	2.772510000
F	4.598650000	-0.363338000	-2.772510000
F	5.273545000	0.565251000	-0.915060000
F	5.273545000	-0.565251000	0.915060000
F	-1.075506000	4.485854000	2.772510000
F	-1.075506000	-4.485854000	-2.772510000
F	-1.091888000	1.502855000	5.050848000
F	-1.091888000	-1.502855000	-5.050848000
F	-1.092029000	5.190111000	-0.915060000
F	-1.092029000	-5.190111000	0.915060000
F	-1.766616000	4.261299000	-2.772510000
F	-1.766616000	-4.261299000	2.772510000
F	-1.766712000	0.574039000	-5.050848000
F	-1.766712000	-0.574039000	5.050848000
F	-2.167201000	4.840767000	0.915060000
F	-2.167201000	-4.840767000	-0.915060000
F	-2.167219000	2.982921000	3.920073000
F	-2.167219000	-2.982921000	-3.920073000
F	-3.506634000	1.139374000	-3.920073000
F	-3.506634000	-1.139374000	3.920073000
F	-3.506822000	2.996965000	-2.772510000
F	-3.506822000	-2.996965000	2.772510000
F	-3.933951000	2.409072000	2.772510000
F	-3.933951000	-2.409072000	-2.772510000
F	-3.934141000	3.557010000	0.915060000
F	-3.934141000	-3.557010000	-0.915060000
F	-4.598634000	2.642414000	-0.915060000
F	-4.598634000	-2.642414000	0.915060000
F	-4.598650000	0.363338000	2.772510000
F	-4.598650000	-0.363338000	-2.772510000
F	-5.273545000	0.565251000	-0.915060000
F	-5.273545000	-0.565251000	0.915060000

30. Perfluoro-C_{60} anion, I_h

C	0.000000000	1.377834000	-3.762969000
C	0.000000000	2.753566000	-2.916057000

C	0.000000000	-1.377834000	3.762969000
C	0.000000000	-2.753566000	2.916057000
C	0.808329000	3.342631000	2.065160000
C	0.808329000	-3.342631000	-2.065160000
C	0.809870000	1.114691000	3.762969000
C	0.809870000	-1.114691000	-3.762969000
C	0.810677000	3.868363000	-0.687253000
C	0.810677000	-3.868363000	0.687253000
C	1.310398000	0.425774000	-3.762969000
C	1.310398000	-0.425774000	3.762969000
C	1.310797000	3.179369000	-2.065160000
C	1.310797000	-3.179369000	2.065160000
C	1.617915000	3.606075000	0.687253000
C	1.617915000	-3.606075000	-0.687253000
C	1.618506000	2.227682000	2.916057000
C	1.618506000	-2.227682000	-2.916057000
C	2.618701000	2.229121000	-2.065160000
C	2.618701000	-2.229121000	2.065160000
C	2.618797000	0.850899000	-2.916057000
C	2.618797000	-0.850899000	2.916057000
C	2.929243000	1.801696000	2.065160000
C	2.929243000	-1.801696000	-2.065160000
C	2.929618000	2.653067000	0.687253000
C	2.929618000	-2.653067000	-0.687253000
C	3.428519000	1.966389000	-0.687253000
C	3.428519000	-1.966389000	0.687253000
C	3.428818000	0.264163000	2.065160000
C	3.428818000	-0.264163000	-2.065160000
C	3.929545000	0.424390000	-0.687253000
C	3.929545000	-0.424390000	0.687253000
C	-0.808329000	3.342631000	2.065160000
C	-0.808329000	-3.342631000	-2.065160000
C	-0.809870000	1.114691000	3.762969000
C	-0.809870000	-1.114691000	-3.762969000
C	-0.810677000	3.868363000	-0.687253000
C	-0.810677000	-3.868363000	0.687253000
C	-1.310398000	0.425774000	-3.762969000
C	-1.310398000	-0.425774000	3.762969000
C	-1.310797000	3.179369000	-2.065160000
C	-1.310797000	-3.179369000	2.065160000
C	-1.617915000	3.606075000	0.687253000
C	-1.617915000	-3.606075000	-0.687253000
C	-1.618506000	2.227682000	2.916057000
C	-1.618506000	-2.227682000	-2.916057000
C	-2.618701000	2.229121000	-2.065160000
C	-2.618701000	-2.229121000	2.065160000
C	-2.618797000	0.850899000	-2.916057000
C	-2.618797000	-0.850899000	2.916057000
C	-2.929243000	1.801696000	2.065160000
C	-2.929243000	-1.801696000	-2.065160000
C	-2.929618000	2.653067000	0.687253000

C	-2.929618000	-2.653067000	-0.687253000
C	-3.428519000	1.966389000	-0.687253000
C	-3.428519000	-1.966389000	0.687253000
C	-3.428818000	0.264163000	2.065160000
C	-3.428818000	-0.264163000	-2.065160000
C	-3.929545000	0.424390000	-0.687253000
C	-3.929545000	-0.424390000	0.687253000
F	0.000000000	1.855949000	-5.047629000
F	0.000000000	3.687380000	-3.920420000
F	0.000000000	-1.855949000	5.047629000
F	0.000000000	-3.687380000	3.920420000
F	1.074963000	4.487308000	2.772218000
F	1.074963000	-4.487308000	-2.772218000
F	1.090899000	1.501494000	5.047629000
F	1.090899000	-1.501494000	-5.047629000
F	1.091762000	5.190232000	-0.915706000
F	1.091762000	-5.190232000	0.915706000
F	1.765112000	0.573520000	-5.047629000
F	1.765112000	-0.573520000	5.047629000
F	1.767910000	4.262156000	-2.772218000
F	1.767910000	-4.262156000	2.772218000
F	2.167388000	2.983153000	3.920420000
F	2.167388000	-2.983153000	-3.920420000
F	2.167488000	4.840707000	0.915706000
F	2.167488000	-4.840707000	-0.915706000
F	3.506907000	1.139463000	-3.920420000
F	3.506907000	-1.139463000	3.920420000
F	3.507237000	2.998461000	-2.772218000
F	3.507237000	-2.998461000	2.772218000
F	3.933995000	3.557264000	0.915706000
F	3.933995000	-3.557264000	-0.915706000
F	3.935502000	2.409005000	2.772218000
F	3.935502000	-2.409005000	-2.772218000
F	4.598831000	2.642197000	-0.915706000
F	4.598831000	-2.642197000	0.915706000
F	4.599865000	0.364304000	2.772218000
F	4.599865000	-0.364304000	-2.772218000
F	5.273577000	0.565543000	-0.915706000
F	5.273577000	-0.565543000	0.915706000
F	-1.074963000	4.487308000	2.772218000
F	-1.074963000	-4.487308000	-2.772218000
F	-1.090899000	1.501494000	5.047629000
F	-1.090899000	-1.501494000	-5.047629000
F	-1.091762000	5.190232000	-0.915706000
F	-1.091762000	-5.190232000	0.915706000
F	-1.765112000	0.573520000	-5.047629000
F	-1.765112000	-0.573520000	5.047629000
F	-1.767910000	4.262156000	-2.772218000
F	-1.767910000	-4.262156000	2.772218000
F	-2.167388000	2.983153000	3.920420000
F	-2.167388000	-2.983153000	-3.920420000

F	-2.167488000	4.840707000	0.915706000
F	-2.167488000	-4.840707000	-0.915706000
F	-3.506907000	1.139463000	-3.920420000
F	-3.506907000	-1.139463000	3.920420000
F	-3.507237000	2.998461000	-2.772218000
F	-3.507237000	-2.998461000	2.772218000
F	-3.933995000	3.557264000	0.915706000
F	-3.933995000	-3.557264000	-0.915706000
F	-3.935502000	2.409005000	2.772218000
F	-3.935502000	-2.409005000	-2.772218000
F	-4.598831000	2.642197000	-0.915706000
F	-4.598831000	-2.642197000	0.915706000
F	-4.599865000	0.364304000	2.772218000
F	-4.599865000	-0.364304000	-2.772218000
F	-5.273577000	0.565543000	-0.915706000
F	-5.273577000	-0.565543000	0.915706000
